# The Cyclopædia of Practical Medicine

**Published:** 1845-03

**Authors:** 


*The Cyclopædia of Practical Medicine.* By Doctors Forbes, Tweedie and Conolly. Edited by Professor Dunglinson. Part XXIV, containing also the title page and index. Philadelphia, Lea & Blanchard.

We congratulate the publishers and the profession on the completion of this important publication. It is seldom that a work of such great intrinsic merit issues from the press; and when we regard its extent, the manner in which it is brought out, and the magnitude of the editors' labours—which have been performed in the midst of numerous other engagements, literary and professional—we cannot but admire the promptitude and punctuality which has been manifested from the beginning of the enterprise.

